# Influence of Mixed Na_2_O/K_2_O on Chemical Durability and Spectral Properties of P_2_O_5_-Al_2_O_3_-BaO-K_2_O-Na_2_O-Nd_2_O_3_ Phosphate Glasses

**DOI:** 10.3390/ma15217439

**Published:** 2022-10-23

**Authors:** Xiben Ma, Yongchun Xu, Jimeng Cheng, Shiyu Sun, Youkuo Chen, Xin Wang, Wei Chen, Shubin Chen, Lili Hu

**Affiliations:** 1 Key Laboratory of Materials for High Power Laser, Shanghai Institute of Optics and Fine Mechanics, Chinese Academy of Sciences, Shanghai 201800, China; 2 Center of Materials Science and Optoelectronics Engineering, University of Chinese Academy of Sciences, Beijing 100049, China

**Keywords:** phosphate glasses, Na/(Na+K) ratios, mixed alkali effect, chemical durability, spectral properties

## Abstract

A series of 56P_2_O_5_-7.5Al_2_O_3_-5.9BaO-(28.56-x)K_2_O-xNa_2_O-1.51Nd_2_O_3_ phosphate glasses with different Na/(Na+K) ratios, which were specially designed for high-power laser application, were prepared by a high-temperature melting method. Except for the density, refractive index, glass transition temperature, and DC conductivity, the chemical durability and spectral properties, as emphasized by high-power and high-energy laser material, were further measured and analyzed. Regarding the chemical durability, the dissolution rates of these glasses do not show an evident mixed alkali effect with increasing the Na/(Na+K) ratio, although the effect is obvious for the glass transition temperature and DC conductivity. To better understand the nature of the dissolution mechanism, the ionic release concentrations of every element are determined. Both Na and K undergo ion exchange, but the ion exchange rate of K is much larger than that of Na. In terms of the spectral properties, the J–O parameters, emission cross-section, radiation lifetime, fluorescence lifetime, effective bandwidth, fluorescence branching ratio, and quantum efficiency are determined from absorption and emission spectra. The trend of Ω_2_ deviating from linearity indicates that the coordination environment symmetry of Nd^3+^ ions and the covalence of Nd-O also present an evident mixed alkali effect. The most important finding is that the emission cross-section and fluorescence lifetime of Nd^3+^ ions at 1053 nm were not affected by the change in the Na/K ratio. According to the above experimental results, the optimized value of the Na/K ratio was determined, based on which the 56P_2_O_5_-7.5Al_2_O_3_-5.9BaO-(28.56-x)K_2_O-xNa_2_O-1.51Nd_2_O_3_ glass maintains a high emission cross-section with good chemical durability.

## 1. Introduction

Nd-doped phosphate glass has become the preferred laser gain medium for large-scale high-power laser systems due to its advantages such as moderate phonon energy, high solubility of rare earth ions, good spectral performance, small nonlinear coefficient, large stimulated emission cross-section, and facile large-scale preparation [1,2,3]. Typical representatives of commercial neodymium glass are the LHG-8 of Hoya company [4], LG-770 of Schott company [5], and N31 neodymium glass of Shanghai Institute of Optics and precision machinery, Chinese Academy of Sciences [6,7]. Generally, neodymium glass consists mainly of (58-62) P_2_O_5_-(8-12) Al_2_O_3_-(12-16) M_2_O-(8-12) MO-(1-2) Nd_2_O_3_. The continuous development of large-scale laser systems has led to higher requirements for laser gain, and the laser gain of neodymium glass is positively correlated with the emission cross-section (σ_ems_) and negatively correlated with the nonlinear refractive index (n_2_). Thus, laser neodymium glass with high σ_ems_ and low n_2_ has become the prime focus in this field of research [7]. The largest difference between high-gain N51 glass and N31 is the decrease in the Al_2_O_3_ molar content, which causes an increase in the emission cross-section of Nd^3+^ ions [8], simultaneously, a decrease in the chemical durability of glass [9,10,11]. Therefore, maintaining the emission cross-section and improving the chemical durability of N51 glass are of significant concern.

The addition of transition metal ions or alkaline earth metals and intermediate oxides [12,13,14] to the glass matrix can improve its chemical durability. However, controlling the chemical durability of glasses without significantly affecting other properties remains a problem. Studies have shown that glasses containing two different types of network modifiers have unusual ionic mobilities [15,16]; this phenomenon is commonly known as the mixed alkali effect (MAE) [17,18,19]. This effect is more significant in conductivity, ion diffusion, chemical durability, glass transition temperature, viscosity, and other characteristics related to mobility, this effect does not cause large structural differences in glasses [20]. The MAE has been studied for improving the chemical durability of simple silicate systems [21,22,23], but research on the chemical durability of phosphate glass is rare [24,25,26]. Yang [24], Fang [25], and others found that the chemical durability of Fe-P glass did not show a distinct mixed alkali effect, whereas Guo et al. [26] showed that the chemical durability of Zn-P glass present an apparent mixed alkali effect. In general, the influence of mixed alkalis on the chemical durability of multicomponent phosphate glass has not been systematically studied.

In this study, we prepared a series of 56P_2_O_5_-7.5Al_2_O_3_-5.9BaO-(28.56-x)K_2_O-xNa_2_O-1.51Nd_2_O_3_ phosphate glasses with different Na/(Na+K) ratios, measured their oxide composition, density, refractive index, glass transition temperature (T_g_), conductivity (σ_dc_), glass dissolution rate (D_r_), ionic release concentrations in solution, infrared transmission spectrum, absorption spectrum, fluorescence spectrum, as well as decay curves, and then systematically studied the influence of MAE on the chemical durability of phosphate glasses and spectral properties of Nd^3+^ ions.

## 2. Experiment Details

### 2.1. Sample Preparation

A series of 56P_2_O_5_-7.5Al_2_O_3_-5.9BaO-(28.56-x)K_2_O-xNa_2_O-1.51Nd_2_O_3_ phosphate glasses were prepared by a high-temperature melting and quenching method. The samples were numbered PKNi (i = 0, 4, 8, 12, 16, 20, 24, 28.56) according to the molar content of Na_2_O present, the starting materials of which were anhydrous reagent powders of Al(PO_3_)_3_, Ba(H_2_PO_4_)_2_, KPO_3_, NaPO_3_, P_2_O_5_, and Nd_2_O_3_. The 500 g of raw materials were fully mixed and poured into a preheated corundum crucible. Furthermore, the corundum crucible was transferred to a silicon carbide rod electric furnace at the melting temperature of 1050 °C for 60 min. After dehydration with CCl_4_ (1050 °C, 1 h) and clarification at a high temperature (1150 °C, 1 h), the glass liquid was poured into a preheated steel mold. Finally, the formed glass was placed in an annealing furnace for 24 h, and the annealing temperature was varied according to the Na/K ratio.

### 2.2. Experimental Method

The annealed glass was processed into 20 mm × 20 mm × 2 mm flakes (polished on both sides) for testing spectral properties and into 14.7 mm × 14.7 mm × 12.1 mm bulk samples (polished on six sides) for hydrolysis. The ion concentrations of P^5+^, Al^3+^, Ba^2+^, Na^+^, K^+^, and Nd^3+^ in the glass samples were determined using an inductively coupled plasma emission spectrometer (ICP-OES), and the values are listed in Table 1.

The density (ρ) was measured with the ELECTRONIC DENSIMETER SD-200L (ALFA MIRAGE, Fukuoka, Japan) instrument. The PRECISON REFRACTOMETER KPR-2000 high-precision refractometer (SHIMADZU, Shimane, Japan) was used to test the refractive index (*n*) according to the V prism method. The glass transition temperature (T_g_) was determined from DSC curve using a differential scanning calorimeter (DSC, sta449/C, Netzsch, Selb, Germany) with a heating rate of 10 °C/min. The conductivity was measured by HIOKI 3522-50lCR testing instrument (HIOKI, Kagoshima, Japan) with experimental test frequency range and the test temperature of 0–100 kHz and 237 °C, respectively. The chemical durability [27,28] was tested by placing bulk samples polished on six sides in a test tube containing 100 mL of high-purity deionized water. After heating in a 90 °C water bath for 48 h, the glass dissolution rate (D_r_) and the ionic release concentrations of each ion in the test tube were measured. The glass dissolution rate (Dr = ∆w/At [26]) was defined as the mass loss per unit surface area and unit time (μg·cm^−2^·h^−1^). The absorption spectrum was measured using the Lambda 950 UV/VIS/NIR spectrophotometer (Perkin−Elmer, Waltham, MA, USA) with a test range of 200–1000 nm and a scan step of 1 nm. The infrared transmission spectrum was measured using a Nicolet FTIR infrared spectrometer (Thermo Scientific, Waltham, MA, USA) between 2000–4000 cm^−1^. An Edinburgh instrument FLSP920 steady-state/transient fluorescence spectrometer was used to test the fluorescence spectrum and lifetime, whereby a Xe-lamp was used as the pump source. The fluorescence spectrum testing range was 850–1500 nm, and the fluorescence lifetime excitation and testing wavelengths were 808 and 1053 nm, respectively.

## 3. Results and Discussion

### 3.1. Density and Refractive Index

**Figure 1:**
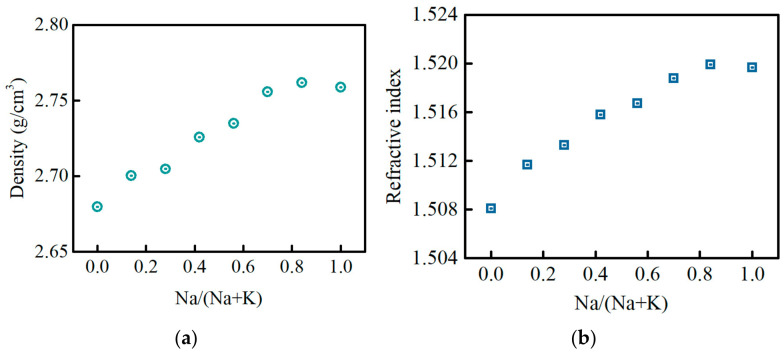
(**a**) Density (ρ) and (**b**) refractive index (*n*) at 656 nm of the PKNi glass with different Na/(Na+K) ratios.

Figure 1a,b exhibits the density (ρ) and the refractive index (n_d_) at 656 nm of the PKNi glass with different Na/(Na+K) ratios, respectively. With an increase in Na/(Na+K) ratio, the ρ and n_d_ gradually increase. However, when Na completely replaces K, the ρ and n_d_ decrease slightly. The Nd^3+^ ion concentration can be calculated by Equation (1):

(1) $$N_{0}=2\rho /M\times w_{t}\%\times N_{A}\;(\mathrm{ions}/{\mathrm{cm}}^{3})$$

where ρ is the density of the glass sample, *M* is the molar mass of the rare earth oxide, *w_t_* is the weight percentage of the rare earth oxide measured by ICP, and *N*_A_ is the Avogadro constant. The Nd^3+^ ion concentration (*N_0_*) and refractive index (n_d_) were used for the subsequent calculation of J–O parameters (Ω_t_) and emission cross-sections (σ_ems_).

### 3.2. Glass Transition Temperature and DC Conductivity

**Figure 2:**
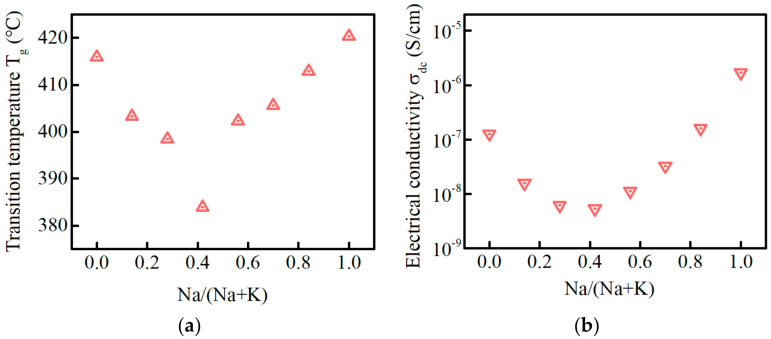
(**a**) Glass transition temperature (T_g_) and (**b**) DC conductivity (σ_dc_) of the PKNi glass at 237 °C, with different Na/(Na+K) ratios.

Figure 2a,b displays the glass transition temperature (T_g_) and the DC conductivity (σ_dc_) of PKNi glass with different Na/(Na+K) ratios, respectively. Glass transition temperature is the temperature corresponding to the transition from glass state to rubbery state. With the increase in Na/(Na+K) ratio, the T_g_ decreases first and then increases, reaching a minimum (383 °C) at Na/(Na+K) = 0.42, as shown in Figure 2a. The T_g_ minimum indicates that there is an MAE in the glass system [19,29].

The MAE is particularly significant in the DC conductivity (σ_dc_), and the DC conductivity can be obtained by fitting the AC conductivity. The DC conductivity (σ_dc_) of glasses with different Na/(Na+K) ratios are shown in Figure 2b. With an increase in the Na/(Na+K) ratio, σ_dc_ decreases first and then increases, reaching a minimum at Na/(Na+K) = 0.42, and PKN28.56 glass is higher than that of PKN0, which is consistent with the observations from Figure 2a. The dynamic structural mismatch model [30,31] can comprehensively explain the change in conductivity of mixed alkali glasses: (1) With the gradual replacement of Na, some K^+^ ions did not migrate successfully, and therefore produced a sharp decline in conductivity. (2) When the Na/(Na+K) ratio was close to 0.5, the number of effective sites were minimal, which resulted in minimal conductivity. (3) When Na/(Na+K) was greater than 0.5, the effective sites of Na^+^ ions began to increase, which was reflected in a sharp increase in the conductivity. Owing to the small radius, the conductivity of PKN28.56 glass was higher than PKN0.

### 3.3. Glass Dissolution Rate and Ion Release Concentration in Solution

The interaction between glass and water will dissolve the glass. It is generally believed that there are two reactions in the dissolution process [32]. (1) Dealkylation reaction: At the glass interface, H^+^ ions in water exchange with alkali metal R^+^ ions on the glass surface, thus triggering the dissolution reaction of glass. A hydration layer will be formed on the glass surface due to the selective dissolution of R_2_O; this process is called the hydration process. (2) Grid dissolution: In the hydration layer on the glass surface, the bridge oxygen bond in the glass structure is constantly attacked by H^+^, and eventually the glass network is destroyed, leading to continuous glass dissolution. The diffusion rate of water molecules in the glass determines the rate of the ion exchange reaction, thereby limiting the formation and development rate of the hydration layer on the glass surface, which is the control step of glass dissolution.

**Figure 3:**
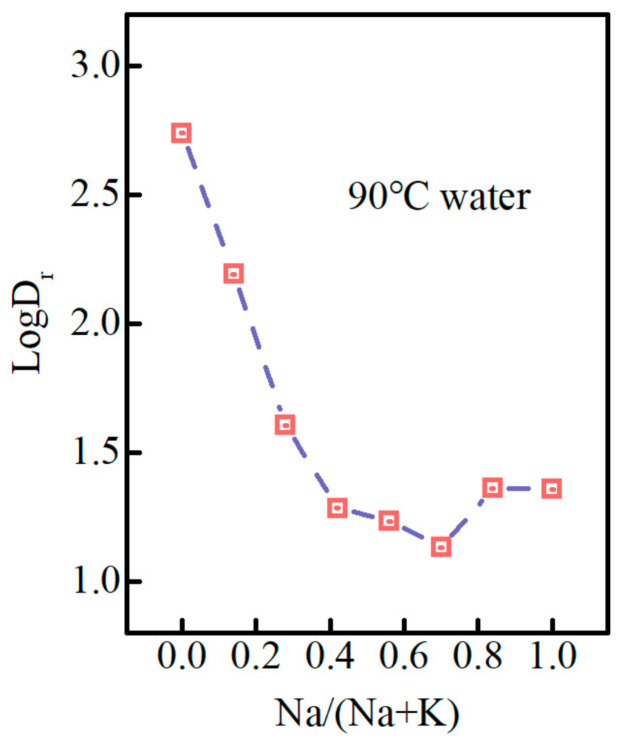
Dissolution rate curve of the PKNi glass with different Na/(Na+K) ratios in a 90 °C water bath for 48 h.

The most studies on the chemical durability of phosphate glass have focused on the glass dissolution rate or ionic release concentration in the solution. Figure 3 shows the glass dissolution rate of PKNi glass with different Na/(Na+K) ratios in a 90 °C water bath for 48 h. The glass dissolution rate (logD_r_) curve is divided into three steps: (1) When 0 ≤ Na/(Na+K) ≤ 0.42, the dissolution rate decreases rapidly, and the logD_r_ decreases from 2.74 to 1.28. (2) When 0.42 ≤ Na/(Na+K) ≤0.7, the logD_r_ starts to decrease slowly from 1.28 to 1.13, reaching the minimum point when Na/(Na+K) = 0.7. (3) 0.7 ≤ Na/(Na+K) ≤ 1, the dissolution rate increases slowly from 1.13 to 1.36. The specific values are listed in Table 2.

**Figure 4:**
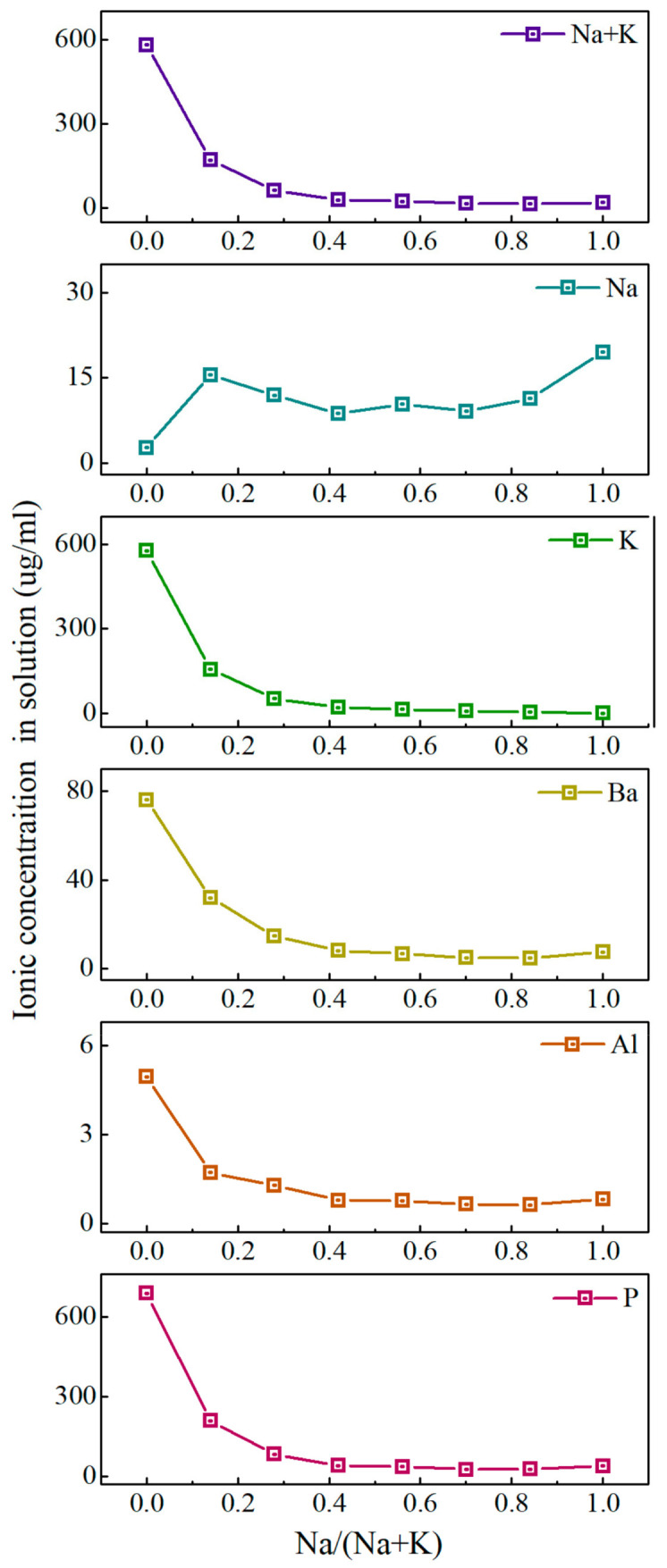
Ionic release concentration in solution of the PKNi glass with different Na/Na+K ratios in a 90 °C water bath for 48 h.

To better understand the release value of each element after the glass was heating in water, we conducted ICP tests on the solution. The ionic release concentration in the solution of the PKNi glass with different Na/(Na+K) ratios in a 90 °C water bath for 48 h is exhibited in Figure 4. When Na/(Na+K) = 0, the ionic release concentrations of P, Al, Ba, and K were the highest, with concentrations of 687, 4.95, 76.14, and 578 μg/mL, respectively. When 0 ≤ Na/(Na+K) ≤ 0.42, the release concentration decreases rapidly, and the values of P, Al, Ba, and K decreased to 41.29, 0.78, 8.13, and 20.16 μg/mL, respectively. When 0.42 ≤ Na/(Na+K) ≤ 1, the ionic release concentration varied slowly. The release concentration of P, which is the glass network former, reached a minimum when Na/(Na+K) = 0.7, while that of Al, which is the glass network intermediate, reached a minimum when Na/(Na+K) = 0.84. The change in Na^+^ ion release concentration is completely different relative to that of other ions, presenting a trend with an initial increase followed a decrease, that then stabilized before finally increasing. The specific values of each ion precipitation concentration are listed in Table 2.

The nature of the dissolution mechanism can be further examined by determining the degree of congruence (DOC) of the glass formers with other elements [33,34]. Based on the original ion concentration in the glass, and the released concentration in the solution after boiling, the DOC is defined as:

(2) $$N_{a}=\frac{number\;cation\;in\;solution}{number\;P^{5+}\;in\;solution}-\frac{number\;cation\;in\;glass}{number\;P^{5+}\;in\;glass}=N_{1}-N_{2}$$

where *N*_a_ is the empirical index of ion exchange overflow, *N*_1_ is the ionic release concentration ratio of cations to *P*^5+^ in the solution, and *N*_2_ is the concentration ratio of cations to *P*^5+^ in the original glass composition. If the ionic release concentration ratio of M^+^/P^5+^ in the solution is the same as that in the original glass composition, namely *N*_a_ = 0, the type of hydrolysis attack is grid breakdown; if the release concentration ratio of M^+^/P^5+^ in the solution is greater than that in the original glass composition, namely *N*_a_ > 0, it is inferred that an additional part is released through ion exchange [34]. Therefore, the cations released in the solution can be categorized into two parts: release by grid breakdown or by ion exchange. Furthermore, *N*_1_ can be expressed as:

(3) $$N_{1}=\frac{number\;cation\;in\;solution(ions-exchange+breakdown)}{number\;P^{5+}\;in\;solution}$$

*N*_b_ is expressed as follows:

(4) $$N_{b}=\frac{N_{1}-N_{2}}{N_{2}}=\frac{number\;cation\;in\;solution(ions-exchange)}{number\;cation\;in\;solution(grid-breakdown)}$$

*N*_b_ is the ratio of cations released through ion exchange to those through grid breakdown (sum is the cations release concentration in the solution).

**Figure 5:**
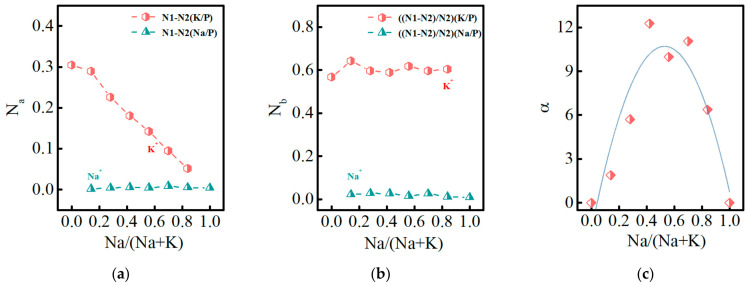
(**a**) *N*_a_ of PKNi glass with different Na/(Na+K) ratios (the empirical index of ion exchange overflow); (**b**) *N*_b_ (ratio of ion exchange and mesh dissolution); (**c**) mixed alkali affect value α.

The *N_a_* and *N_b_* trends of Na^+^ and K^+^ ions for PKNi glasses with different Na/(Na+K) ratios are shown in Figure 5a,b. When Na/(Na+K) = 0, the *N_a_* value of K^+^ ion is 0.30. As Na^+^ started to replace K^+^, the *N_a_* values of K^+^ and Na^+^ were 0.29 and 0.002, respectively. With the increase in the Na/(Na+K) ratio, the *N_a_* of K^+^ ions decreased continuously, while the Na^+^ ions increased slowly. This indicates that the Na^+^ and K^+^ ions in the glass continuously underwent ion exchange as ion exchange rate of K^+^ is much larger than that of Na^+^, which is related to the high ion field strength of Na^+^. It can be observed from Figure 5b that with an increase in the Na/(Na+K) ratio, the *N_b_* values of K^+^ and Na^+^ ions fluctuate at approximately 0.6 and 0.02, respectively, indicating that the unit [PO_4_] grid dissolution will be accompanied by a 0.6-unit K^+^-H^+^ and 0.02-unit Na^+^-H^+^ ion exchange, which has a weak relationship with the concentration of Na^+^ and K^+^ ions.

The value *α* is defined as the extent of MAE:

(5) $$\alpha =\frac{A-B}{B}$$

(6) $$A=a_{1}M_{1}+a_{2}M_{2}$$

where *a*_1_ and *a*_2_ represent the dissolution coefficients of Na and K (calculated when the alkali metals in the glass were Na and K, *a*_1_ = 0.7 and *a*_2_ = 19.67), respectively. *M*_1_ and *M*_2_ represent the molar content of Na and K (ICP data), respectively. A and B represent the theoretical and actual ionic release concentration values of the total alkali metal in the solution, respectively. Using the above formula, the α of MAE plotted in Figure 5c. When Na/(Na+K) = 0.42, the MAE is most evident, which is consistent with the trends of conductivity and T_g_.

Therefore, we divided the phosphate glass system with different Na/(Na+K) ratios into three sections for discussion: (1) When 0 ≤ Na/(Na+K) ≤ 0.42, some K^+^ ions in the grid are replaced by Na^+^ ions. Since the ion exchange rate and grid dissolution rate of Na are much smaller than those of K, and the MAE gradually increases, the glass dissolution rate decreases rapidly and chemical durability increases. (2) When 0.42 ≤ Na/(Na+K) ≤ 0.7, Na^+^ ions gradually begin to dominate. However, the MAE is the strongest in the ratio range, resulting in a slow decrease in the dissolution rate of the glass. (3) When 0.7 ≤ Na/(Na+K) ≤ 1, Na+ ions dominate in the grid and the MAE gradually weakens, resulting in a slight increase in the glass dissolution rate.

### 3.4. Absorption Spectrum

**Figure 6:**
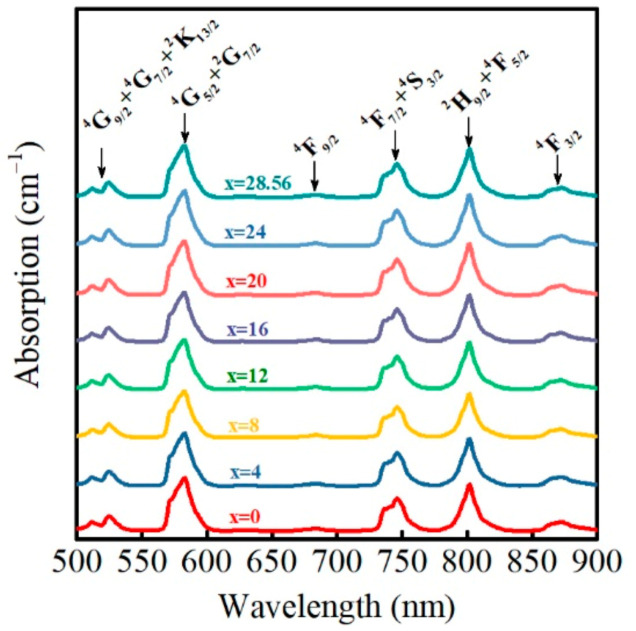
Absorption spectrum of Nd^3+^ ions in PKNi glass with different Na/(Na+K) ratios between 500–900 nm.

The absorption spectra of Nd^3+^ ion in PKNi glass with different Na/(Na+K) ratios are shown in Figure 6. The absorption at 523, 582, 684, 746, 801, and 870 nm corresponds to the transitions from ^4^I_9/2_ to (^4^G_9/2_+^4^G_7/2_+^2^K_13/2_), (^4^G_5/2_+^2^G_7/2_), (^4^F_9/2_), (^4^F_7/2_+^4^S_3/2_), (^2^H_9/2_+^4^F_5/2_), and ^4^F_3/2_, respectively [35,36,37]. According to the Judd–Ofelt (J–O) theoretical model [38,39], the J–O parameters were calculated by standard least square fitting of the experimental oscillator strength (*f_exp_*) and calculated oscillator strength (*f_cal_*). The experimental oscillator strength (*f_exp_*) can be calculated by Equation (7) [40]:

(7) $$f_{\exp}=\frac{2.303m_{e}c^{2}}{\pi e^{2}{\bar{\lambda }}^{2}N_{0}l}\int OD\left(\lambda \right)d\left(\lambda \right)$$

where *m_e_* and *e* are the mass and charge of an electron, respectively, *c* is the speed of light, is the central wavelength, *N_0_* is the ion concentration of Nd^3+^, *l* is the thickness of the glass sample, and OD (λ) is the optical density. The calculated oscillator strength *f_cal_* from the initial *J* state to the final *J’* state is calculated using Equation (8):

(8) $$f_{cal}\left(J;J^{\prime }\right)=\frac{8\pi^{2}mc}{3h\left(2J+1\right)\bar{\lambda }}\left[\frac{{\left(n^{2}+2\right)}^{2}}{9n}S_{ed}\left(J;J^{\prime }\right)+nS_{md}\right]$$

(9) $$S_{ed}\left(J;J^{\prime }\right)=\sum_{t=2,4,6}\Omega_{t}{\left|\left(S,L\right)JU^{t}\left(S^{\prime },L^{\prime }\right)J^{\prime }\right|}^{2}$$

(10) $$S_{md}\left(J;J^{\prime }\right)={\left(\frac{h}{4\pi mc}\right)}^{2}{\sum_{t=2,4,6}\left|\left(S,L\right)J\vec{L}+2\vec{S}\left(S^{\prime },L^{\prime }\right)J^{\prime }\right|}^{2}$$

where *h* is the Planck constant, *n* is the glass refractive index, 2*J*+1 is the degeneracy of the originating level of the transition, *S_ed_* and *S_md_* are the electric and magnetic dipole line strengths, respectively,|*(S,L)JU^t^(S′,L′)J′*|^2^ is the doubly reduced matrix elements of the tensor transition operator, which is determined by rare earth elements and unrelated to the host material [41], and Ω_t_ (t = 2,4,6) represents the J–O parameters. The values of Ω_t_ in PKNi glass with different Na/(Na+K) ratios are given in Table 3.

The J–O parameter (Ω_2_) reflects the symmetry of the rare earth ion coordination environment and covalency of the Nd–O bonds, while Ω_4_ and Ω_6_ reflects the glass rigidity. The larger the Ω_2_, the lower the symmetry of the Nd^3+^ ion coordination environment and the greater the covalency of the Nd–O bonds. When Na/(Na+K) = 0, Ω_2_ has the maximum value of 3.79 × 10^−20^ cm^2^, which indicates that the Nd^3+^ ion coordination environment symmetry is the lowest and the covalency of the Nd–O bonds is the strongest in PKN0 glass. With an increase in the Na/(Na+K) ratio, Ω_2_ shows an initially increasing and then decreasing trend, reaching a minimum point when Na/(Na+K) ratio is 0.56. When Na/(Na+K) = 1, Ω_2_ is 3.14 × 10^−20^ cm^2^. Moreover, Ω_2_ deviates from the linear trend, indicating that the symmetry of the Nd^3+^ ion coordination environment and the covalence of the Nd-O bonds have an obvious MAE. However, the values of Ω_4_ and Ω_6_ do not change significantly with different Na/(Na+K) ratios, indicating that the glass rigidity is basically not affected by the mixed alkali conditions.

### 3.5. Infrared Transmittance and Fluorescence Lifetime

**Figure 7:**
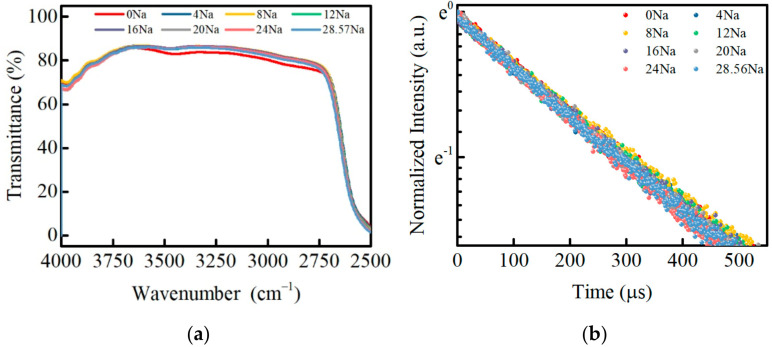
(**a**) Infrared transmission spectra; (**b**) fluorescence decay curve of Nd^3+^ ion at 1053 nm in PKNi glasses with different Na/(Na+K) ratios.

Figure 7a shows the infrared (IR) transmission spectrum of the PKNi glass with different Na/(Na+K) ratios. A higher hydroxyl absorption coefficient enhances the non-radiative transitions of the upper energy level and affects the lifetime of rare earth ions, which influences the luminescence performance of laser glass. The absorption coefficient of hydroxyl OH^-^ is determined by the following Equation (11):

(11) $$\alpha \left(OH^{-}\right)=log\left(T_{0}/T\right)/L$$

where *T*_0_ refers to the maximum infrared transmittance of the glass, *T* is the transmittance at 3000 cm^−1^, and *L* represents the thickness of the sample. The values of α*(OH^−^)* of these glass samples are less than 0.2 cm^−1^, indicating that the whole group of glass exhibits beneficial water removal properties.

Figure 7b shows the fluorescence decay curve of Nd^3+^ ion at 1053 nm in PKNi glasses with different Na/(Na+K) ratios. With an increase in the Na/(Na+K) ratio, the fluorescence decay curves of the Nd^3+^ ions almost coincide, and do not change significantly, indicating that the MAE has little effect on the fluorescence lifetime. The fluorescence intensity of the Nd^3+^ ions generally conform to single exponential decay, and the fluorescence lifetime of PKNi glasses with different Na/(Na+K) ratios obtained by fitting are given in Table 3.

### 3.6. Fluorescence Properties

**Figure 8:**
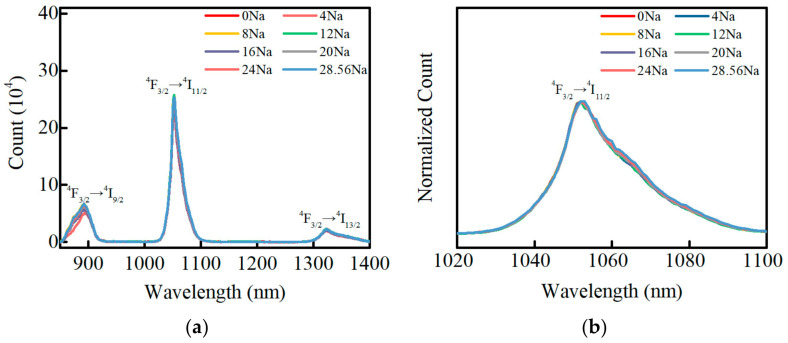
(**a**) Fluorescence spectra of Nd^3+^ ions in PKNi glasses with different Na/(Na+K) ratios in the range of 850–1450 nm; (**b**) Normalized fluorescence spectrum at 1053 nm (808 nm excitation).

The fluorescence spectra of the Nd^3+^ ions in PKNi glasses with different Na/(Na+K) ratios are shown in Figure 8a. Excited by the Xe-lamp at 808 nm, the Nd^3+^ ions in the ground state ^4^I_9/2_ are first pumped to the excited state energy levels ^4^F_5/2_ and ^2^H_9/2_, then undergo non-radiative transition to the ^4^F_3/2_ level, and finally undergo radiative transition to the ^4^I_9/2_, ^4^I_11/2_, and ^4^I_13/2_ levels, corresponding to the fluorescence peaks at 892, 1053, and 1330 nm, respectively. With an increase in Na/(Na+K) ratio, the intensities of the fluorescence peaks at 892, 1053, and 1330 nm did not change significantly. Figure 8b presents the normalized fluorescence spectrum of Nd^3+^ ions at 1053 nm. The full width at half maxima (FWHM) of the Nd^3+^ ions at 1053 nm changes slightly with an increase in Na/(Na+K) ratio and the effective bandwidth (Δλ_eff_) can be obtained from the normalized fluorescence spectrum.

According to the J–O parameter (Ω_t_) obtained from the previous fitting, the radiation transition probabilities (A) from ^4^F_3/2_ to ^4^I_9/2_, ^4^I_11/2_, and ^4^I_13/2_ can be obtained by Equation (12) [42]:

(12) $$A\left(J\to J^{\prime }\right)=\frac{64\pi^{4}e^{2}}{3h\lambda^{3}(2J+1)}[\frac{n{\left(n^{2}+2\right)}^{2}}{9}S_{ed}]$$

where *J* = 3/2 and *J’* = 9/2, 11/2, and 13/2, respectively, and λ is the central wavelength. The fluorescence branching ratios (*β*) are the ratios of the radiative transition probabilities to the sum of all radiative transition probabilities, which can be calculated using Equation (13):

(13) $$\beta_{J\to J\prime }=\frac{A_{J\to J\prime }}{\sum A}$$

The stimulated emission cross-section (*σ_ems_*) from ^4^F_3/2_ to ^4^I_11/2_ can be determined according to Equation (14):

(14) σems=λ4A(F3/24→I11/24)8πcn2Δλeff

where *c* is speed of light, *n* is the glass refractive index, *λ* is the peak wavelength of emission, and *Δλ_eff_* is the effective bandwidth, which can be obtained using Equation (15):

(15) $$\Delta \lambda_{eff}=\frac{\int I(\lambda )d\lambda }{I_{\max}}$$

where *I(λ)* represents the luminescence intensity at wavelength λ, and *I_max_* is the maximum intensity.

The radiation lifetime (*τ_rad_*) can be calculated using Equation (16):

(16) $$\tau_{rad}=\frac{1}{\sum J^{\prime }A_{\left(J\to J^{\prime }\right)}}$$

The quantum efficiency (*η*) can be determined via Equation (17):

(17) $$\eta =\frac{\tau_{f}}{\tau_{rad}}$$

The radiation transition probabilities (A), fluorescence branching ratios (β), stimulated emission cross-section (σ_ems_), effective bandwidth (Δλ_eff_), radiation lifetime (τ_rad_), and quantum efficiency (η) of Nd^3+^ ions at 1053 nm are listed in Table 3. In the range of 0.28 ≤ Na/Na+K ≤ 0.84, the glass has a smaller radiation lifetime and larger quantum efficiency.

**Figure 9:**
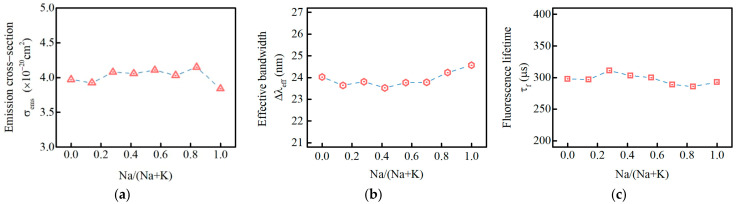
(**a**) Stimulated emission cross−section (σ_ems_); (**b**) effective bandwidth(Δλ_eff_); (**c**) fluorescence lifetime (τ_f_) of the Nd^3+^ ions at 1053 nm in PKNi glass with different Na/(Na+K) ratios.

The stimulated emission cross-section, fluorescence lifetime are important parameters for evaluating laser performance. The stimulated emission cross-section (σ_ems_), the effective bandwidth (Δλ_eff_), and the fluorescence lifetime (τ_f_) of the Nd^3+^ ions at 1053 nm in PKNi glass are shown in Figure 9. With an increase in the Na/(Na+K) ratio, the σ_ems_, Δλ_eff_, and τ_f_ show an initially increasing and then decreasing trend, an initially decreasing and then increasing trend, and an initially rising and then decreasing trend, respectively; however, the overall difference in the range is negligible. The laser neodymium glass for high-power laser systems requires a large σ_ems_ and long τ_f_, while in the range of 0.28 ≤ Na/(Na+K) ≤ 0.84, Nd^3+^ ions have a large emission cross-section and fluorescence lifetime.

### 3.7. Optimized Matrix Glass When Na/Na+K = 0.7

The dissolution rate D_r_ of PKNi glass with Na/(Na+K) = 0.7 was 13.542 μg·cm^−2^·h^−1^ (listed in Table 2), which was 1/40 that of PKN0 glass (549.167 μg·cm^−2^·h^−1^), and depended on the low ion exchange rate of Na^+^-H^+^, the low [PO_4_] grid dissolution rate, and the strong MAE. The glass size of this experiment and LHG-8 used for chemical durability test [43] were 14.7 mm × 14.7 mm × 12.1 mm and 26 mm × 26 mm × 12 mm, respectively, and deionized water temperature were 90 °C and 50 °C, respectively. Although the test temperature of PKNi glasses was higher than that of LHG-8 [43], the dissolution rate, D_r_, of PKNi glass with Na/(Na+K) = 0.7 was close to that of the LHG-8 neodymium glass, which was 20.833 μg·cm^−2^·h^−1^ [43]. As is well known, LHG-8 is a very good commercialized Nd laser glass material, which is widely used in high-energy and high-power laser systems. By the mixed alkali effect, the dispersion rate of PKNi glass was successfully adjusted to the same level as that of LHG8. Therefore, the chemical durability of PKNi glass with Na/(Na+K) = 0.7 should be guaranteed in practical laser applications.

On the other hand, the J–O parameters Ω_2_, Ω_4_, and Ω_6_ of PKNi glass with Na/(Na+K) = 0.7 were 3.12 × 10^−20^ cm^2^, 5.67 × 10^−20^ cm^2^, and 4.91 × 10^−20^ cm^2^, respectively, while those of LHG-8 were 4.4 × 10^−20^ cm^2^, 5.1 × 10^−20^ cm^2^, and 5.6 × 10^−20^ cm^2^, respectively. There are obvious differences between both, which indicate that the local environment of Nd^3+^ ion in the two kinds of matrix glasses is obviously different. This is mainly because PKNi glass reduces both P and Al content, making Nd^3+^ ions have better spectral parameters. The emission cross-sections (σ_ems_) of PKNi glass with Na/(Na+K) = 0.7 and LHG-8 were 4.03 × 10^−20^ cm^2^ and 3.6 × 10^−20^ cm^2^ [8], respectively. LHG-8 glass has been successfully applied in laser fusion drivers such as the National Ignition Facility and Laser Megajoule. When Na/(Na+K) = 0.7, the PKNi glass with excellent chemical durability has a higher σ_ems_ than the LHG-8, indicating that it has sufficient laser output capacity.

A series of studies on the Na/K ratio in 56P_2_O_5_-7.5Al_2_O_3_-5.9BaO-(28.56-x)K_2_O-xNa_2_O-1.51Nd_2_O_3_ glass greatly improve the chemical durability and spectral characteristics of N51 glass. Although N51 Nd-doped phosphate laser glass is still under further improvement before scale production, N51 has been successfully trialed in liquid-cooled KJ-class laser applications [44].

## 4. Conclusions

The influences of the mixed alkali effect (MAE) on the chemical durability and spectral properties of Nd^3+^ ions in 56P_2_O_5_-7.5Al_2_O_3_-5.9BaO-(28.56-x)K_2_O-xNa_2_O-1.51Nd_2_O_3_ phosphate glasses were studied systematically. With an increase in Na/(Na+K) ratio, the glass dissolution rate and ionic release concentration in the solution show an initial rapid decreasing trend, then decreased slowly, and lastly, increased slightly, depending on the combined action of the ion exchange rate, [PO_4_] grid dissolution rate, and mixed alkali effect. With an increase in Na/(Na+K) ratio, the Ω_2_ related to the symmetry of Nd^3+^ ions and covalent nature of the Nd–O bonds initially decreased and then increased, while the Ω_4_ and Ω_6_, related to glass rigidity, did not change significantly. The emission cross-section (σ_ems_) fluctuates between 3.89 × 10^−20^ cm^2^ and 4.11 × 10^−20^ cm^2^, and the lowest value is still higher than that of LHG-8 commercial glass. The radiation lifetime (τ_rad_), fluorescence lifetime (τ_f_), effective bandwidth (Δλ_eff_), fluorescence branching ratios (β), and quantum efficiency (η) do not change significantly with Na/Na+K ratio.

Above all, it is proved experimentally that the 56P_2_O_5_-7.5Al_2_O_3_-5.9BaO-(28.56-x)K_2_O-xNa_2_O-1.51Nd_2_O_3_ neodymium glass with Na/(Na+K) = 0.7 has two main advantages as necessitated by high-power and high-energy laser material. Its dissolution rate regarding chemical durability is relatively low (13.542 μg·cm^−2^·h^−1^), meanwhile its emission cross-section regarding spectral property is reasonably large (4.03 × 10^−20^ cm^2^).

## Figures and Tables

**Table 1:** Chemical composition (mol%) of PKNi glasses with different Na/K ratios measured by ICP.

Sample	P_2_O_5_	Al_2_O_3_	BaO	K_2_O	Na_2_O	Nd_2_O_3_	O/P Ratio
PKN0	55.03	7.60	5.82	29.54	0.3	1.69	3.07
PKN4	55.12	8.32	5.94	24.88	4.01	1.73	3.09
PKN8	55.96	7.80	5.86	21.17	7.75	1.45	3.06
PKN12	55.82	8.06	5.88	17.16	11.51	1.57	3.07
PKN16	55.87	8.21	5.85	12.90	15.61	1.56	3.07
PKN20	56.33	7.78	5.91	8.93	19.34	1.71	3.06
PKN24	56.63	7.43	5.97	4.80	23.52	1.62	3.04
PKN28.56	57.21	7.27	5.98	0.07	27.88	1.59	3.03

**Table 2:** Dissolution rate and ion release concentration in solution of PKNi glass with different Na/(Na+K) ratios.

Sample	Dissolution Rate D_r_ (μg·cm^−2^·h^−^^1^)	logD_r_	P^5+^ (μg/mL)	Al^3+^ (μg/mL)	Ba^2+^ (μg/mL)	K^+^ (μg/mL)	Na^+^ (μg/mL)
PKN0	549.167	2.74	687.7	4.95	76.14	578.5	2.74
PKN4	158.833	2.19	209.4	1.72	32.03	155.2	15.56
PKN8	40.210	1.60	84.28	1.29	14.71	50.9	11.99
PKN12	19.167	1.28	41.29	0.78	8.13	20.16	8.74
PKN16	17.083	1.23	36.67	0.77	6.81	13.69	10.4
PKN20	13.542	1.13	25.91	0.65	4.97	6.56	9.12
PKN24	22.917	1.36	27.07	0.63	4.85	3.68	11.37
PKN28.56	22.710	1.36	39.72	0.82	7.51	0	19.52

**Table 3:** The J–O parameter, emission cross-section, radiation lifetime, fluorescence lifetime, radiation transition probabilities (A), effective bandwidth, fluorescence branching ratios, quantum efficiency of Nd^3+^ ion at 1053 nm in PKNi glasses with different Na/(Na+K) ratios.

Sample	Ω_2_ (10^−20^ cm^2^)	Ω_4_ (10^−20^ cm^2^)	Ω_6_ (10^−20^ cm^2^)	σ_ems_ (10^−20^ cm^2^)	τ_rad_ (μs)	τ_f_ (μs)	∑A_rad_ (s^−1^)	Δλ_eff_ (nm)	β (%)	η (%)
PKN0	3.79	5.7	4.92	3.97	364	298	2750	24.02	48.5	82
PKN4	3.47	5.51	4.78	3.93	372	297	2685	23.65	48.5	80
PKN8	3.38	5.78	4.99	4.08	355	311	2819	23.81	48.5	88
PKN12	3.26	5.66	4.9	4.06	360	303	2780	23.53	48.5	84
PKN16	2.95	5.82	4.99	4.11	351	300	2849	23.77	48.5	85
PKN20	3.12	5.67	4.91	4.03	357	289	2802	23.79	48.5	81
PKN24	3.24	5.95	5.14	4.15	340	286	2943	24.23	48.5	84
PKN28.56	3.14	5.57	4.83	3.84	362	293	2759	24.57	48.5	81

## Data Availability

The data that support the findings of this study are contained within the article.
